# Chemicals from *Agave sisalana* Biomass: Isolation and Identification

**DOI:** 10.3390/ijms16048761

**Published:** 2015-04-20

**Authors:** Jener David Gonçalves Santos, Ivo Jose Curcino Vieira, Raimundo Braz-Filho, Alexsandro Branco

**Affiliations:** 1 Laboratório de Fitoquímica, Departamento de Saúde, Universidade Estadual de Feira de Santana, Feira de Santana, Bahia 44031-460, Brazil; E-Mail: jennerdgs@yahoo.com.br (J.D.G.S.); 2 LCQUI-CCT, Universidade Estadual do Norte Fluminense, Campos dos Goytacazes, Rio de Janeiro 28013-600, Brazil; E-Mails: curcino@uenf.br (I.J.C.V.); braz@uenf.br (R.B.-F.); 3 LCQUI-CCT, Universidade Estadual do Norte Fluminense Darcy Ribeiro, Campos dos Goytacazes-RJ/DEQUIM-ICE, Universidade Federal Rural do Rio de Janeiro, Seropédica, Rio de Janeiro 23894-374, Brazil; E-Mail: rbraz@ufrrj.br (R.B.-F.)

**Keywords:** *Agave sisalana*, sisal, biomass, chemicals

## Abstract

*Agave sisalana* (sisal) is known worldwide as a source of hard fibers, and Brazil is the largest producer of sisal. Nonetheless, the process of removing the fibers of the sisal leaf generates 95% waste. In this study, we applied chemical sequential steps (hydrothermal extraction, precipitation, liquid-liquid extraction, crystallization, SiO_2_ and Sephadex LH 20 column chromatography) to obtain pectin, mannitol, succinic acid, kaempferol and a mixture of saponins as raw chemicals from sisal biomass. The structural identification of these compounds was performed though spectrometric methods, such as Infrared (IR), Ultraviolet (UV), Mass spectrometry (MS) and Nuclear magnetic resonance (NMR). All the sisal chemicals found in this work are used by both the chemical and pharmaceutical industries as excipients or active principles in products.

## 1. Introduction

The *Agave sisalana* is an important plant to semi-arid regions worldwide due to its commercial applications as a supplier of hard fibers for the production of wires and cords [1,2]. In 2011, the world production of sisal fibers exceeded 410,000 tons. Out of this total, 283,000 tons were produced in Brazil, representing more than 69% of world production [3]. These fibers furnish between 4% and 5% of the fresh weight, and the remainder, considered waste, is composed of water, parenchymal tissue, short fibers, polysaccharide, inorganic compounds [4,5] and secondary metabolites, such as steroidal saponins [6].

Consequently, the growth of both the crop and the agro-industrial production observed in recent decades has caused increased discard [7]. That represents a problem, which is not only economic, but also environmental. It is also a moral challenge for modern society [8]. Moreover, other factors are associated with the increase in agro-industrial discard, such as: characteristics of plants, obtaining process, perishable products, limited information related to waste, and others [9].

The use of technologies that reduce the negative environmental impacts of the agricultural production is thought to be important for the protection of natural resources for generations to come [10]. The methods of waste treatment are selected based on the composition or the specificity of the residue. Among the various treatment processes, the most commonly used are thermal processes, evaporation, composting, combustion, anaerobic digestion, anaerobic digestion and co-transesterification of coagulation [11]. The techniques used for the waste treatment can affect the physical, chemical or biological characteristic of the waste, reducing its volume and/or toxicity, or even making it safer.

The agro-industrial and food sectors are examples of the implementation of strategies aimed at the efficient and cost-effective recycling of waste [12,13]. Thus, a waste material is considered to be valued as a raw material for generating new products when the residue has been treated as a byproduct of the production process. For example, grape pomace residues could be used as a source of phenolic compounds, such as anthocyanins, flavonoids, flavonol glycosides and phenolic acids [14]. Obtaining extracts or supply of bioactive products of high added value, such as flavors, antioxidants, cosmetics, drugs or drug adjuvants, reveals a potential opportunity to the agro-industrial waste industry [15,16,17].

**Figure 1:**
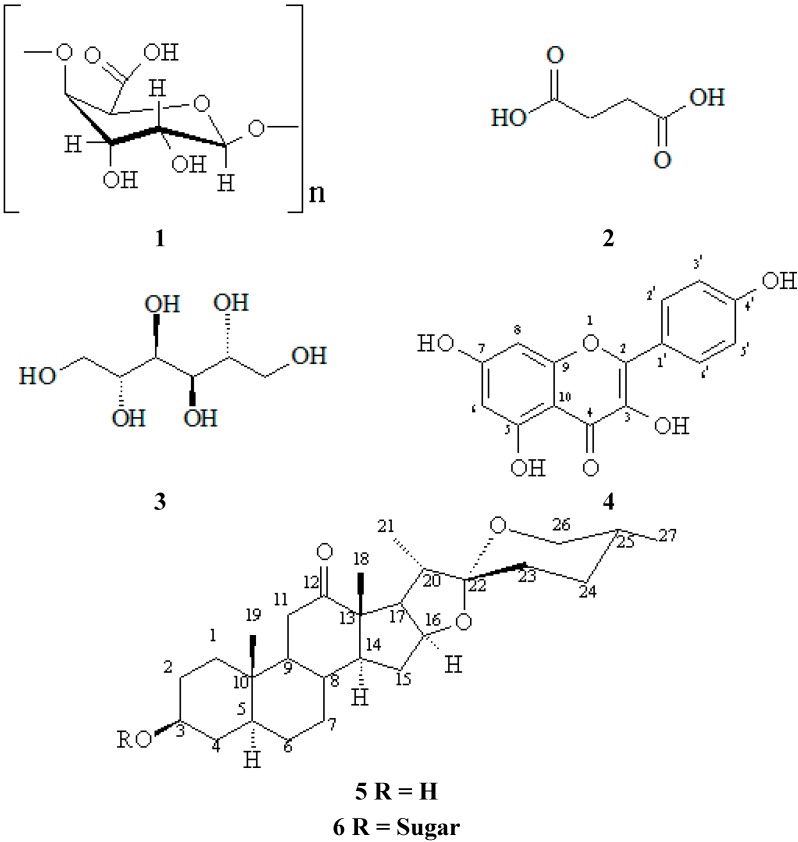
Chemical structure of the value-added compounds isolated from sisal waste: Pectin (**1**), Mannitol (**2**), Acid succinic (**3**), Kaempferol (**4**), Hecogenin (**5**) and Saponins (**6**).

The utilization of Brazilian sisal waste is still largely untapped, despite its indication for use as an organic fertilizer supplement and feed for ruminants, as well as the supply of feedstock for the production of corticosteroids [18,19]. Some studies led by our research group describe the use of sisal waste to obtain extracts with biological activities such as larvicidal [20], antimicrobial [21], ovicidal [22] and antiparasitic [23]. In this study, we show the application of the chemical procedures to isolate chemicals (Figure 1) from *A. sisalana* waste with a future possibility to generate added value to the sisal agroindustry.

## 2. Results and Discussion

### Isolation and Characterization of Chemicals from Sisal Biomass

**Figure 2:**
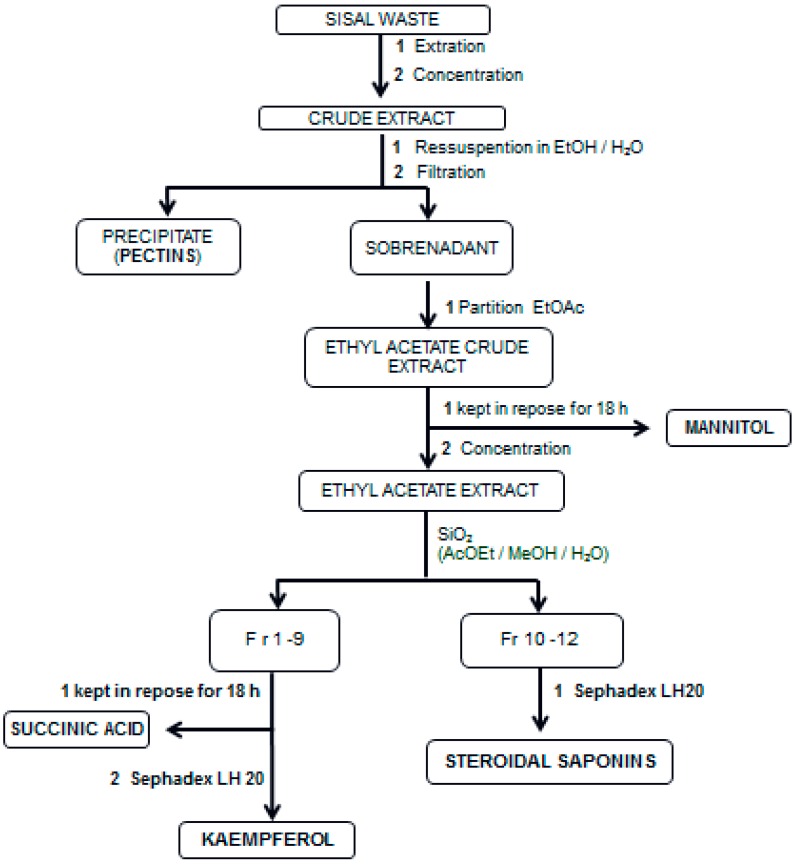
Scheme to obtain chemicals from sisal biomass.

Figure 2 shows the chemical procedures applied in sisal biomass for the isolation of its chemicals. The literature described five recovery stages for components of high added value from agro-industrial wastes: macroscopic pre-treatment, macro- and micro-molecules separation, extraction, isolation and purification and product formation [12].

In general, *Agave* species contains about 20% non-structural carbohydrate [24]. That explains the use of these carbohydrates in the production of beverages from the stems of various *Agave* spp., including the sweet drink aguamiel (fermented pulp), distilled mescal, and tequila. Tequila, made from *Agave tequilana*, is of major importance as both a domestic and export product from Mexico [25]. In sisal waste, a significant amount of polysaccharides was also found. The first step in our phytochemical procedures was the removal of a significant amount of carbohydrates (83%) through precipitation [26], which yielded an easy-to-handle (e.g., chromatographic column) and easy-to-characterize (spectroscopic analysis) sisal extract.

The infrared spectra of the precipitate show bands at ν 3441 cm^−1^ (OH); 2986 cm^−1^ (CH_2_ groups) and 1617 and 1431 cm^−1^, which corresponds to vibrations of the O=C–O structure. These data may imply that the precipitate is composed mainly of pectin (**1**) in comparison with data from the commercial pectin [27].

After the removal of polysaccharide from the hydroalcoholic extract, the liquid-liquid partition with ethyl acetate was submitted. This system was set aside at room temperature for 24 h. After this time, many crystals were formed and characterized as mannitol (**2**). Mannitol has already been described in literature by our group from sisal waste produced in Brazil [28].

After the removal of pectin and mannitol, the ethyl acetate extract was fractionated on an open column packed with silica gel, which allowed the isolation of other chemicals (See Figure 1). Compound **3** showed ^1^H NMR spectrum with signal δ_H_ 12.15 ppm characteristic for carboxylic acids. The ^13^C NMR spectrum showed two signals at δc 173.8 and 28.9, indicating the presence of dicarboxylic acid. Furthermore, this compound was subjected to esterification reaction with isobutanol to confirm the presence of the carboxylic acid grouping structure. Compound **3** was characterized as succinic acid, a product of the tricarboxylic acid cycle.

**Figure 3:**
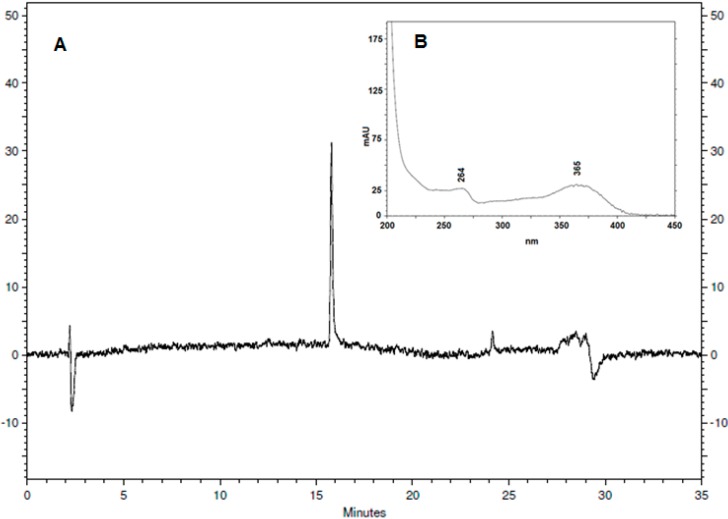
(**A**) Chromatogram of flavonoid from sisal waste obtained through HPLC-DAD (365 nm); (**B**) UV spectra of peak at 15.7 min.

The HPLC-DAD analysis of compound **4** at a wavelength at λ_max_ 365 nm showed only one peak with a retention time (RT) at 15.7 min. This peak showed characteristic absorptions in UV spectra of the flavonoid compounds (Figure 3). The ^1^H NMR spectrum revealed two sets of *meta*-coupled doublets at δ_H_ 6.09 (1H, *J* = 2.1 Hz) and 6.30 (1H, *J* = 2.1 Hz), and the signals were assigned to H-6 and H-8, respectively. The presence of a set of AA'BB' doublets at δ_H_ 6.83 (2H, *J* = 9.0 Hz) 8.04 (2H, *J* = 9.0 Hz), each integrating with two hydrogens, has been assigned to H-2'/H-6' and H-3'/H-5', respectively.

The HSQC spectrum of **4** shows the correlation between the signals: δ_H_ 8.05 and δ_C_ 129.5; δ_H_ 6.94 and δ_C_ 116.0 proposed for the carbons CH-2'/CH-6' and CH-3'/CH-5', respectively. Other correlations showed interaction in the ring A with signals at δ_H_ 6.44 and δ_C_ 93.2; δ_C_ 98.5 and δ_H_ 6.19 [29]. These results allowed the identification of **4** as flavonol kaempferol.

In 1989, researchers isolated a mixture of saponins from the sisal juice cultivated in São Paulo (Brazil), which they named as sisalins [30]. These saponins showed the identical sapogenin (hecogenin, **5**) and different sequences on the sugar position in the glycoside chain of each one. On the other hand, studies with the dried fermented residues of leaf-juices and with the fresh leaves from *A. sisalana* have found new saponins with other sapogenins [6,31,32].

**Figure 4:**
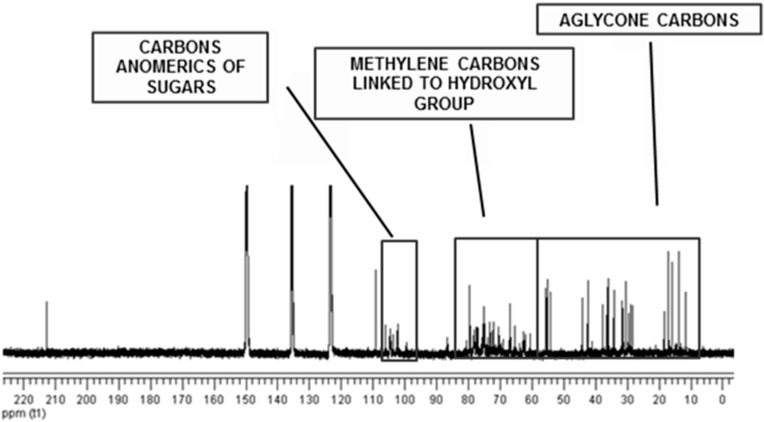
^13^C NMR spectrum of saponin-rich fraction (C_5_D_5_N, 125 MHz).

In the study showed here, we also obtain the sisalin complex according to some compounds described by Ujikawa and Purchio [30]. The ^13^C NMR spectrum (Figure 4) showed intense signal at δc 109.3 ppm, which was attributed to cetal carbon in C-22 characteristic of spirostan saponins [33]. The carbon signals between δ_C_ 10 and 60 ppm referred to the steroidal structure; 60 and 80 ppm were linked to carbon atoms with OH, sugars and a covalent bond between carbon and oxygen of the aglycone; δ_C_ 100 and 106 were characteristic to carbon hemiacetalics (anomeric) for the sugar units present in saponins. The signal δ_C_ 212.8 indicates carbonyl C-12 in its structure.

The analysis of the DEPTQ-13C NMR spectrum allowed the recognition of the presence of 27 signals attributed to hecogenin (**5**) in steroidal saponins (Table 1). The above analyses showed hecogenin as the only aglycone of the saponin-rich fraction obtained from sisal waste, which seems to be linked to the sugar moieties producing different glycosidic chains.

**Table 1:** ^1^H (500 MHz) and ^13^C (125 MHz) NMR data for **5** including results obtained by heteronuclear 2D shift-correlated HSQC (Heteronuclear Single Quantum Correlation) and HMBC (Heteronuclear Multiple Quantum Correlation), in pydine-*d*_5_ as solvent and Tetramethylsilane (TMS) used as internal reference. Chemical shifts (δ, ppm) and coupling constants (*J*, Hz, in parenthesis). *

Position	HSQC		HMBC		Model	
**C**	**δ_C_**	**δ_H_**	**^2^*J*_HC_**	**^3^*J*_HC_**	**δ_C_**	**δ_H_**
10	36.0	-	3H-19	-	36.3	-
12	212.6	-	-	-	212.8	-
13	55.1	-	3H-18	-	55.5	-
22	109.2	-	-	3H-21	109.2	-
**CH**	**δ_C_**	**δ_H_**	**^2^*J*_HC_**	**^3^*J*_HC_**	**δ_C_**	**δ_H_**
3	76.9	3.83	-	H-1'	76.7	3.88 (*m*)
5	44.2	0.84	-	3H-19	44.3	0.86 (*m*)
8	34.1	1.73	-	-	34.4	1.65
9	55.2	0.89	-	3H-19	55.3	0.92
14	55.6	1.35	-	3H-18	55.8	1.38
16	79.5	4.58	-	-	79.6	4.99 (*m*)
17	54.2	2.75 (*t*, 8.5)		3H-18; 3H-21	54.2	2.76 (*dd*)
20	42.4	1.90	3H-21	-	42.5	1.96
25	30.3	1.54	-	-	30.4	1.57
**CH_2_**	**δ_C_**	**δ_H_**	**^2^*J*_HC_**	**^3^*J*_HC_**	**δ_C_**	**δ_H_**
1	36.1	1.35, 0.70	-	3H-19	36.6	1.36, 0.76
2	29.4	1.55, 1.40	-	-	29.6	2.00, 1.76
4	34.4	1.76, 1.32	-	-	34.1	1.90, 1.78
6	28.3	1.10	-	-	28.5	1.16
7	31.5	1.69, 1.64	-	-	31.7	1.63, 0.78
11	37.7	2.35 (*t*, 14.5), 2.22 (*dd*, 14.5, 5.0)	-	-	37.9	2.41 (*dd*, 13.8, 13.7) 2.25 (*dd*, 13.8, 5.0)
15	31.2	2.10, 1.58	-	-	31.4	2.10, 1.61
23	31.4	1.55	-	-	31.6	1.69 (*m*), 2H
24	29.0	1.98	-	3H-27	29.1	1.56 (*m*), 2H
26	66.7	3.58 (*dd*, 10.5), 3.48 (*t*, 105)		3H-27	66.9	3.59 (*br d*, 11.8) 3.48 (*dd*, 11.8, 10.6)
**CH_3_**	**δ_C_**	**δ_H_**	**^2^*J*_HC_**	**^3^*J*_HC_**	**δ_C_**	**δ_H_**
18	15.9	1.07 (*s*)	-	-	16.0	1.07 (*s*)
19	11.5	0.64 (*s*)	-	-	11.8	0.88 (*s*)
21	13.7	1.34 (*d*, 7.0)	-	-	13.8	1.35 (*d*, 6.9)
27	17.1	0.68 (*d*, 6.0)	-	-	17.2	0.69 (*d*, 5.7)

* Number of hydrogens bound to carbon atoms assessed through comparative analysis of DEPTQ-^13^C NMR spectra. Chemical shifts and coupling constants (*J*) obtained from tra. 1D ^1^H NMR spectrum. Superimposed ^1^H signals are described without multiplicity and chemical shifts deduced through HSQC, HMBC and ^1^H-^1^H-COSY spectra.

## 3. Experimental Section

### 3.1. General Procedures

Ethyl acetate, butanol, ethanol and methanol (analytical grade) from VETEC were used. Analytical thin-layer chromatography (TLC) was performed on commercial aluminum plates coated with silica gel (0.025 mm) (Merck, Darmstadt, Germany). Spots were visualized by spraying with 1 M H_2_SO_4_ and heating to 100 °C. Silica gel (Kielselgel 60, 70–230 mesh) was used for open-column chromatography. *iso*Butanol and concentrated sulfuric acid was used as the organic acid in the esterification reaction.

Fourier transform-infrared (FT-IR) spectra were obtained in Perkin Elmer FTIR Spectrometer 100 (Norwalk, CT, USA) with KBr film (Sigma-Aldrich, St. Louis, MO, USA). NMR spectra were obtained using a Bruker AC-500 (^1^H: 500 MHz; ^13^C: 125 MHz, Bruker, Billerica, MA, USA). HSQC and HMBC spectra were recorded on a Bruker Avance DRX-500 (500 MHz, Bruker, Billerica, MA, USA). Chemical shifts are expressed in ppm, and coupling constants (*J*) are measured in Hertz (Hz). Pyridine (C_5_D_5_N) was used as a solvent with tetramethylsilane as the internal reference.

The analysis of high-performance liquid chromatography coupled to photodiode detector (HPLC-DAD) (EZChrom Elite, Darmstadt, Germany) equipped with VRW HITACHI L 2130 pump and HITACHI L-2300 VRW diode array detector was obtained from the Merck-Hitachi^®^LaChron Elite chromatograph (Darmstadt, Germany). The results were acquired and processed using EZChrom Elite software, injection volume of 20 μL. In this process, we used a LiChrospher^®^ column (C-18 reverse phase; 5 mm, 250 × 4.6 mm, Darmstadt, Germany). Samples and the mobile phase were filtered through cellulose acetate membranes with pore size of 0.22 mm. The samples and standard sample were dissolved in methanol.

### 3.2. Obtaining Crude Extract from Sisal Waste

The sisal waste was collected after the process of decortication of the sisal leaves, on a sisal farm located in Valente, in the state of Bahia (S 11°24'53.4"), in May 2008. Seventy kilograms of sisal waste was refluxed with distilled water (70 L) for three hours. After this procedure, the extract was filtered and concentrated to yield 6.1 kg (8.7%) of crude extract.

### 3.3. Systematic Chemical Procedures on Crude Extract

The 6.1 kg of crude extract was resuspended in 12 L of ethanol/water solution (8:2) and kept at room temperature for 18 h to remove the polysaccharides though precipitation. After this time, the precipitate was filtrated and hydroalcoholic extract (without polysaccharides) was partitioned with ethyl acetate (2:3, *v*/*v*) and the system maintained at rest for 24 h. The formation of crystals was observed in the organic phase and characterized as mannitol. The remaining was filtered and concentrated in a rota-evaporator to yield 363 g (0.51%) of ethyl acetate extract (EAE).

Fifty milligrams of the EAE was subjected to open-column chromatography packed with silica gel, eluted with a gradient of solvents (ethyl acetate, methanol and water) resulting in 16 fractions. Fractions 7 (EtOAc; 100%, 200 mL) and 8 (EtOAc/MeOH, 8:2, *v*/*v*, 200 mL) furnished crystal characterized as succinic acid (2.9 g). After that, these fractions were united and subjected to a fractionation through gel permeation chromatography on a glass column packed with Sephadex LH 20 column eluted with methanol giving 10 fractions (1a to 10a). After the TLC analysis, flavonoid was identified in fraction 8a. The fractions, Fr 10 (EtOAc/MeOH 40:60, 100 mL), Fr 11 (EtOAc/MeOH, 20:80, 100 mL), Fr 12 (MeOH 100%, 100 mL) and Fr 13 (MeOH/H_2_O, 8:2, 100 mL), were united and subjected to fractionation by Sephadex LH 20 eluted with methanol yielding 12 fractions (1b–12b). The fractions 7, 8 and 9b were positive for saponins through TLC analysis and were united to furnish the steroidal saponins in the mixture (sisalins).

### 3.4. HPLC-DAD Analysis

Flavonoid was analyzed under the following conditions: mobile phase to two-phase system: A (0.1% H_3_PO_4_ in H_2_O-acidified water) and B (methanol) gradient: time 0–20 min. (75% A and 25% B to 100% B); 20–24 min. (100% B); 24–25 min. (75% A and 25% B); and 25–35 min. (75% A and 25% B). Reading at 280 nm.

## 4. Conclusions

In this work, it was possible to obtain sequentially from sisal waste: pectin, applied in the food industry by providing increased viscosity and playing a role as a stabilizer in foods and beverages; mannitol, used as chiral ligands and chiral-building blocks; succinic acid, used as raw material for the synthesis of various products such as those produced through condensation and polymerization reactions; and flavonoids and steroidal saponins, natural compounds with several biological activities. The application of this proposal for the use of sisal waste can be an initial step to evaluate the economic and environmental viability for the implementation of a pilot scale and future industrial application.
